# Cost-effectiveness analysis of single use negative pressure wound therapy dressings (sNPWT) compared to standard of care in reducing surgical site complications (SSC) in patients undergoing coronary artery bypass grafting surgery

**DOI:** 10.1186/s13019-018-0786-6

**Published:** 2018-10-03

**Authors:** Leo M Nherera, Paul Trueman, Michael Schmoeckel, Francis A Fatoye

**Affiliations:** 1 Smith & Nephew Advanced Wound Management, Global Market Access, 101 Hessle Road, Hull, HU3 2BN UK; 2 Vascular and Diabetic Centre Department of Heart Surgery, Asklepios Klinik St. Georg Cardiac, Lohmühlenstr 5, 20099 Hamburg, Germany; 3 Department of Health Professions, Manchester Metropolitan University, Manchester, UK

**Keywords:** Single use negative pressure wound therapy; cost-effectiveness, Surgical site complications, CABG

## Abstract

**Background:**

There is a growing interest in using negative pressure wound therapy in closed surgical incision to prevent wound complications which continue to persist following surgery despite advances in infection measures.

**Objectives:**

To estimate the cost-effectiveness of single use negative pressure wound therapy (sNPWT) compared to standard of care in patients following coronary artery bypass grafting surgery (CABG) procedure to reduce surgical site complications (SSC) defined as dehiscence and sternotomy infections.

**Method:**

A decision analytic model was developed from the Germany Statutory Health Insurance payer’s perspective over a 12-week time horizon. Baseline data on SSC, revision operations, length of stay, and readmissions were obtained from a prospective observational study of 2621 CABG patients in Germany. Effectiveness data for sNPWT was taken from a randomised open label trial conducted in Poland which randomised 80 patients to treatment with either sNPWT or standard care. Cost data (in Euros) were taken from the relevant diagnostic related groups and published literature.

**Results:**

The clinical study reported an increase in wounds that healed without complications 37/40 (92.5%) in the sNPWT compared to 30/40 (75%) patients in the SC group *p* = 0.03. The model estimated sNPWT resulted in 0.989 complications avoided compared to 0.952 and the estimated quality adjusted life years were 0.8904 and 0.8593 per patient compared to standard care. The estimated mean cost per patient was €19,986 for sNPWT compared to €20,572 for SC resulting in cost-saving of €586. The findings were robust to a range of sensitivity analyses.

**Conclusion:**

The sNPWT can be considered a cost saving intervention that reduces surgical site complications following CABG surgery compared to standard care. We however recommend that additional economic studies should be conducted as new evidence on the use of sNPWT in CABG patients becomes available to validate the results of this economic analysis.

## Background

There has been advances in infection control practices and wound dressings yet surgical site infections (SSI) remains common in patients undergoing surgery [1–4]. European Centre for Disease Prevention and Control (ECDC) reports that SSI are among the most common healthcare-associated infections (HAIs) which occur after surgery in the area of the body where the surgery took place. European-wide SSI incidence rates range from 0.7% in knee prosthesis to 9.7% in colon surgery [2]. Reddy et al. [3], reported that approximately 0.3–5% of median sternotomy incisions are affected by complications, such as infection and dehiscence.

Surgical site infections impacts on morbidity, health- related quality of life, longer post-operative hospital stays, additional surgical procedures, mortality and increased costs [4–10]. Graf et al. [6] estimated the financial loss to a hospital due to deep sternal wound infection following coronary artery by-pass surgery to be $12,482 (€9154) in Germany. In the United Kingdom, attributable median hospital length of stay (LOS) due to SSI for cardiac patients is estimated to be 23 days and the attributable median costs due to SSI are £11,003 ($8517- $15,395) respectively [10].

Many strategies have been introduced to control SSI, ranging from antibiotics prophylaxis, dressings and new protocols including the use of single use negative pressure wound therapy (sNPWT). Evidence on the clinical effectiveness of sNPWT is accumulating rapidly [11, 14, 15] and has been shown to be effective in reducing SSI in closed incisions such as in caesarean-section, orthopaedic and cardiac surgery. Cost-effectiveness studies have been performed in patients undergoing caesarean-section [13] and Orthopaedic surgery [12], however the cost-effectiveness of sNPWT following cardiothoracic surgery has not been reported.

This study therefore examined the cost-effectiveness of sNPWT PICO^◊^ (Smith & Nephew, Hull, UK) compared with standard care dressing (standard post-operative dressings) in preventing surgical site complications defined as dehiscence and sternotomy infections in patients undergoing coronary artery by-pass (CABG) surgery from a Germany Insurance payer’s perspective.

## Methods

**Fig. 1:**
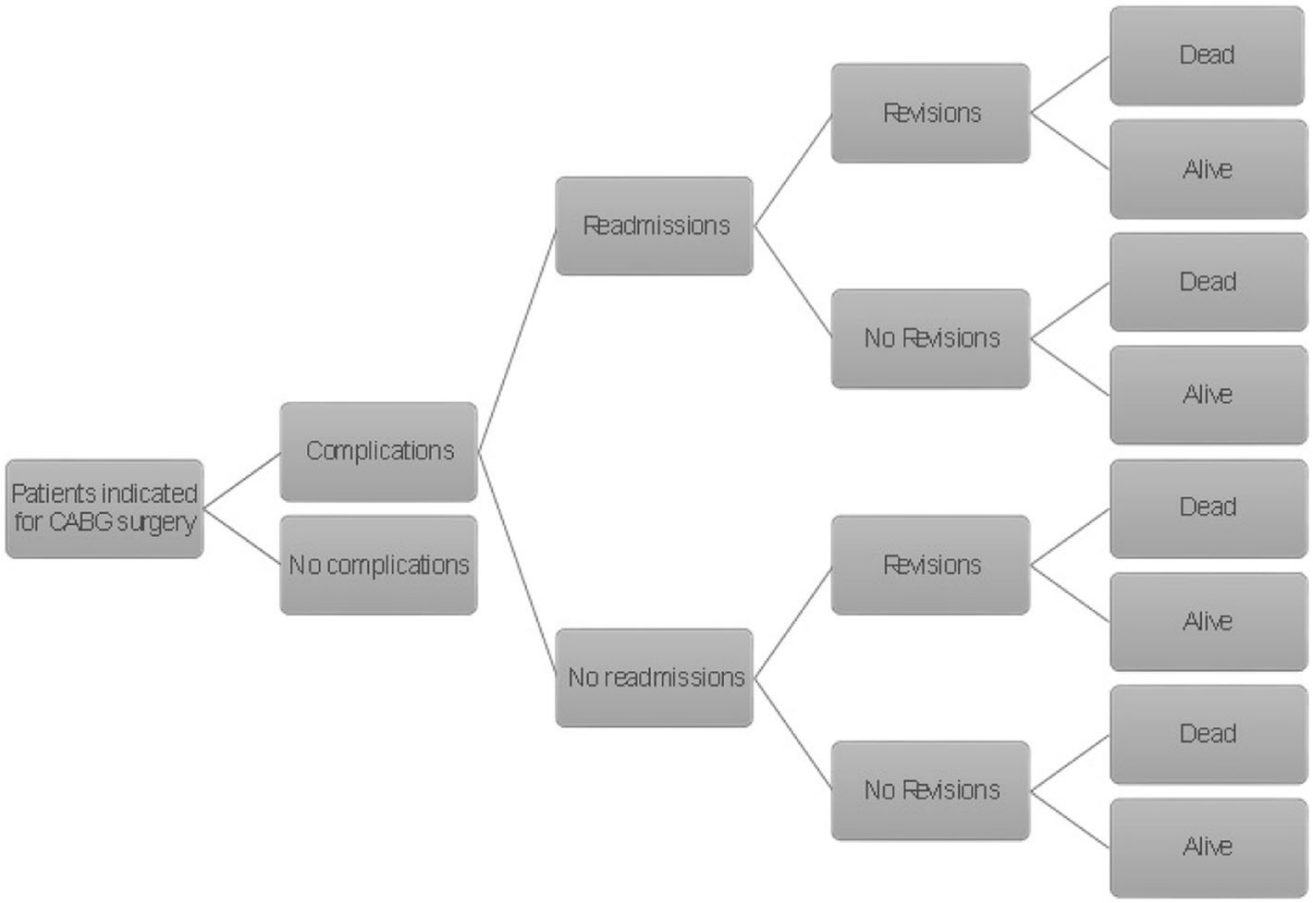
Model structure for sNPWT compared to SC in patients following CABG surgery. The decision tree model used to predict cost and outcome of sNPWT and standard of care. The tree maps the outcomes (health states) modelled following a complication or no complication. The branches for no complication are not shown in the figure

To describe the clinical problem, we constructed a decision analytic model in Microsoft Excel 2016 (Microsoft Corporation, Redmond, WA, US) to simulate the expected outcomes and costs of patients undergoing CABG surgery. The mean age of patients that were modelled is 65 years, which represents the mean age of the majority of patients included in the studies were baseline and effectiveness data were drawn from [16, 17]. Following skin closure, one group would be managed by sNPWT while the other group would receive standard of care dressings. The modeled patients may develop complications of sternotomy wounds which in this model was defined as SSI (superficial and deep wound infections) or dehiscence. The complications are assumed to result in readmissions and revision surgery in some cases as shown in Fig. 1. The model assumed a proportion of patients could die from natural causes and also die due to surgery. The perspective adopted was that of the Statutory Health Insurance payers in Germany. The economic model adopted a 12 week time horizon to enable the both superficial and deep infection to manifest themselves. Superficial SSI usually occurs within 30 days after surgery while deep SSI normally occurs within 30 to 90 days following surgery [1]. No discounting was done for both costs and outcomes due to a shorter time horizon (12 weeks). The schematic representation of the model is shown below in Fig. 1, showing the branches of the complications node, there are similar branches for the no complications node not shown in the figure.

### Baseline clinical data

Data for this economic analysis were derived from published clinical studies. In particular the baseline data was obtained from a single centre prospective observational study that followed patients who underwent CABG for 36 months in Germany [16]. The study collected information on the following outcomes; revision operations, patients’ length of stay, and readmissions to the hospital from 2621 patients. Twenty-seven patients (4.85%) were diagnosed SSI according to the Centres for Disease Control and Prevention criteria. Data on length of stay, readmission, revision surgery and mortality due to surgery was taken from the same source [16].

Effectiveness data for sNPWT was taken from a randomised open label trial conducted in Poland [17]. The study evaluated sNPWT use in patients after an off-pump CABG procedure, using the internal mammary artery in 80 patients. There were 40 patients in each arm with similar patient characteristics ie, 40 in sNPWT and 40 in the standard care arm in whom conventional dressings were applied in the postoperative period. The ECDC definition of SSI was used in this study. The endpoint of the study was wound healing defined as absence of SSI and wound dehiscence of wound margins without clinical or microbiological signs of infections. 37/40 (92.5%) patients had their wounds healed without complications in the sNPWT compared to 30/40 (75%) patients in the standard care group *p* = 0.03 [17]. We calculated the Odds ratio (OR) from this data to be, OR;0.22, 95% confidence interval 0.06 to 0.81, *p* = 0.002 We are also aware of an ongoing study in Spain which is comparing sNPWT with standard of care in patients undergoing CABG with preliminary results expected in 2019 (Dr Carlos Velasco, Hospital Juan Canalejo, Spain; − personal communication).

Strugala and Martin found that sNPWT reduced length of stay on average by 0.5 days [14] in a meta-analysis that assessed the prophylactic use of PICO negative pressure wound therapy on surgical site complications. We applied this reduction in the base case model and assessed the assumption that there was no difference in sensitivity analysis. All-cause mortality was obtained from the Germany Federal Statistical Office [18]. We made a further assumption that mortality following revision surgery will be 30% higher than that of patients who did not have revision surgery in accordance to published literature [19]. The clinical data used in the model is shown in Table 1.

**Table 1:** Clinical and utility data used in the model for sNPWT compared to standard care in patients following CABG surgery. The table shows the baseline data, effectiveness of sNPWT and health related quality of life (utility) data that was applied in the model

Outcome	Mean	Number of patients	Events	Distribution	Reference
Baseline SSC rate	0.048	2621	127	Beta	Cristofolini [16]
Mortality with SSC	0.017	118	2	Beta	ibid
Mortality without SSC	0.007	2503	18	Beta	
Readmission SSC	0.034	118	4	Beta	
Readmission No SSC	0.000	2503	1	Beta	
Revision SSC	0.068	118	8	Beta	
Revision No SSC	0.005	2503	12	Beta	
All-cause mortality	0.003				[18]
Multiplier for revision mortality	1.300				Wu [19]
Length of stay data					
Length of stay with surgical site complications	Mean	Lower CI	Upper CI		
Intensive care unit	15.2	1	87.2	Log normal	Cristofolini [16]
Intermediate care	4.8	0.5	25.2	Log normal	
General ward	22.3	0.5	68.4	Log normal	
Length of stay without surgical site complications					
Intensive care unit	3.8	1	26	Log normal	Cristofolini [16]
Intermediate care	2.4	0.5	10	Log normal	
General ward	8.3	0.5	19	Log normal	
Utility data used in the model					
Parameter	Mean	Alpha	Beta	Distribution	Source
Disutility with SSI	0.2	8	41	Beta	Tuffaha [13]
Utility with no SSI	0.91	185	18	Beta	
Effectiveness data (Odds ratio and 95% CI)					
Outcome	Mean	Lower CI	Upper CI	Distribution	Source
Odds ratio for SSC	0.220	0.060	0.810	Log normal	Witt-Majchrzak [17]
Reduction in LOS (days)	0.500	0.020	0.70	Log normal	Strugala [14]

Abbreviations: *sNPWT* single use negative pressure wound therapy, *SSC* surgical site complications, *CI* confidence interval, *LOS* length of stay

### Health state utilities

The health state utilities in the model were sourced from published literature. The utility scores for patients undergoing CABG and discharged without complications were set at 0.91 and for those discharged with complications was set at 0.71 obtained from a study by Tuffaha 2015. The study by Tuffaha et al. considered the cost-utility analysis of negative pressure wound therapy in high-risk caesarean section wounds [13]. Currently there is no evidence that utility values differ by type of dressing used, we therefore assumed that utility was independent of the type of dressing in the model. The utility data parameters used in the model is shown in Table 1.

### Cost data

Costs were derived from standard cost references with resource utilisation valued in Euros (2017). For inpatient care we used data from Cristofolini who identified length of stay in different hospital wards from intensive care unit to the general ward before discharge for patients with or without infections. We calculated the mean cost for a patient with or without infection and applied it in the model. The cost for the stay in each ward were obtained from the hospital management website [20]. For procedure costs, we used the average reimbursement costs from the relevant Germany Diagnosis Related Group Report Browser 2017 of the procedure code 5–361 “Application of an aortocoronary bypass”, and Procedure code 5–363.1: “Revision of an aortocoronary bypass” see Table 2. The mean cost for the main procedure was estimated to be €15,135.58, while for revision it was estimated to be €24,740.45. We assumed that costs of standard of care dressings and nursing costs were all included in the DRG costs while the cost of the intervention (sNPWT) was obtained from the manufacturer. The model applied the cost of one sNPWT device, which is designed to last for 7 days and is supplied with two dressings. In the sensitivity analysis we assumed patients received two sNPWT dressings to assess changes in expected total costs.

**Table 2:** Cost data used in the model for sNPWT compared to standard care in patients following CABG surgery

Cost component	Mean cost	Lower value	Upper value	Source
Cost of hospital stay in ICU ward (inclusive of all done inpatient)	€1400.00	€1050.00	€1750.00	[20]
Intermediate ward	€850.00	€637.50	€1062.50	ibid
General ward cost/day	€200.00	€150.00	€250.00	
CABG procedure (code 5–361.^a^: “Application of an aortocoronary bypass”)	€15,135.58	€11,351.69	€18,919.48	ibid
Cost of revision CABG procedure	€24,740.45	€18,555.34	€30,925.56	ibid
Outpatient rehabilitation	€1726.46	€1294.85	€2158.08	[21]
Community doctor consultation fee per quarter	€16.53	€12.40	€20.66	[22]
Electrocardiography	€16.53	€12.40	€20.66	ibid
Community Cardiologist	€21.06	€15.80	€26.33	ibid
Duplex-Electrocardiography	€71.50	€53.63	€89.38	ibid
Home visits	€11.53	€8.65	€14.41	ibid
sNPWT unit cost	€153.00	€114.75	€191.25	^a^

Abbreviations: *CABG* coronary artery bypass grafting, *sNPWT* single use negative pressure wound therapy, Gamma distribution was used for costs, we assumed the cost values will be 25% above and below the mean value to calculate the lower and upper values. ^a^Data obtained from manufacturer

For post discharge outpatient consultations, we assumed patients would be seen in an outpatient rehabilitation facilities for 3 weeks. Rehabilitation costs were obtained from a study by Zeidler et al. [21] which considered cost of outpatient and inpatient rehabilitation for cardiac diseases in Germany. Mean costs were inflated to 2018 using the Germany consumer price index and were estimated to be €1726.47 for outpatient rehabilitation which included the costs of 25–30 min of physiotherapy once per week for 3 weeks. In addition, we included the costs of one community doctor and community cardiologist visit where an electrocardiography a duplex-electrocardiography would be done respectively. Furthermore, the cost of once a week visit by a community nurse for 6 weeks was also estimated [22]. Cost of post-surgery medication was assumed to be the same ie, patients were all prescribed antiplatelet, statins, beta-blockers, ACE inhibitors and was therefore not explicitly costed. The relevant costs and their sources are presented in Table 2.

### Cost-effectiveness and sensitivity analysis

The incremental cost-effectiveness ratio is the added cost per additional unit of health, in this model measured in quality adjusted life years (QALYs) and complications avoided. This was calculated as the difference between the expected costs divided by the expected difference between the QALYs or complications avoided of sNPWT and standard care over the modelled time horizon.

Sensitivity analysis was done to assess the uncertainty around the model inputs and their impact on the main conclusions of the model. One-way sensitivity analyses were conducted by varying some of the critical model parameters, each key parameter was alternately assigned a low and high value then re-evaluate the cost-effectiveness results. Furthermore, we implemented a probabilistic sensitivity analysis where we assigned prior distributions to model parameter and then simultaneously selecting values at random from those distributions using Monte Carlo simulation to estimate the expected costs and effects associated with each intervention. The lognormal distribution was implemented to capture the uncertainty surrounding the treatment effect; the gamma and beta distributions were used to capture the uncertainty in cost and utility values respectively.

## Results

The total mean costs per patient in the sNPWT group were lower than the total mean costs per patient in the standard care group. The use of sNPWT was associated with more QALYs and fewer wound related complications compared to the use of standard care. Overall, the use of sNPWT is a dominant strategy (cost-saving) compared to standard care as it costs less and results in better clinical outcomes as shown in Table 3.

**Table 3:** Base case results for sNPWT compared to standard care in patients following CABG surgery

Intervention	Costs	Complications avoided	QALYs	Cost difference	Complication difference	QALY difference
Standard of care	€ 20,572	0.952	0.8593			
sNPWT	€ 19,986	0.989	0.8904	-€ 586	0.0374	0.0311

Abbreviations: *sNPWT* single use negative pressure wound therapy, *QALY* quality adjusted life years

### Sensitivity analysis

One-way sensitivity analyses was performed and the results are displayed in Table 4 showing the mean incremental costs of sNPWT compared to standard care. Negative costs shows that sNPWT is cost-saving compared to standard care, hence model’s conclusions were not changed by changes in input parameters tested as shown in Table 4 where all the cost differences are below €0.

**Table 4:** Results of one way-sensitivity analysis for sNPWT compared to standard care in patients following CABG surgery

Parameter, mean value (lower and upper value)	Savings with lower value	Savings with upper value
Baseline Risk 4.8% (2.5% and 7.8%)	€ 428	€ 793
Treatment effect complications 0.22 (0.06 and 0.81)	€ 654	€ 337
Cost of sNPWT €153 (€114.75 and €191.25)	€ 624	€ 548
Number of sNPWT 1 (2)	€ 433	
Length of stay difference 0.5 (0)	€ 178	

Abbreviations: *sNPWT* single use negative pressure wound therapy

**Fig. 2:**
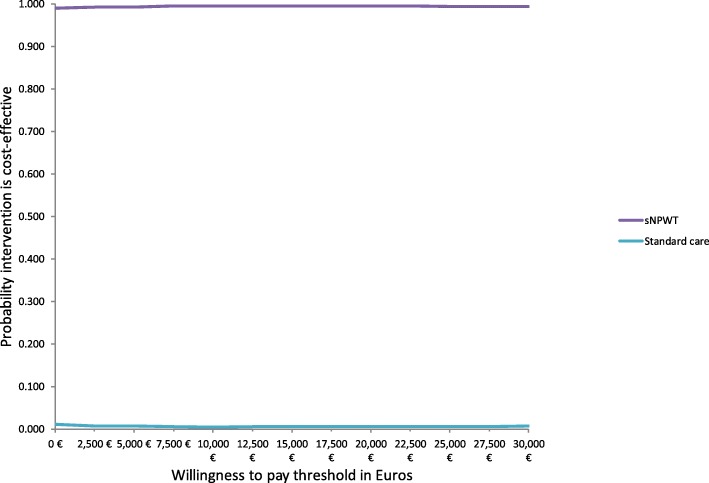
Cost effectiveness acceptability curves for sNPWT compared to standard care in patients following CABG surgery. Cost-effectiveness acceptability curves depicting results of the probabilistic sensitivity analysis for the two interventions sNPWT and SC. The y-axis gives the probability that each intervention is cost effective as a function of willingness to pay shown on the x-axis. A willingness to pay of €50,000/QALY is within the bounds of accepted cost-effectiveness thresholds. The figure suggests there is little uncertainty regarding the cost-effectiveness of sNPWT compared to SC (100% probability that sNPWT is cost-effective)

**Fig. 3:**
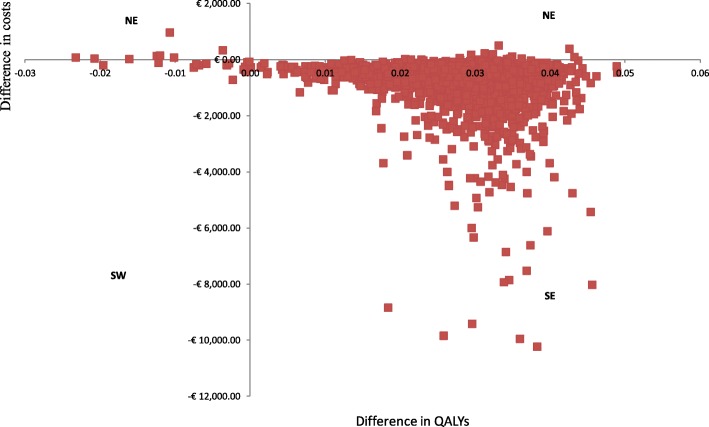
Cost-effectiveness plane for sNPWT compared to standard care in patients following CABG surgery. The cost-effectiveness plane shows results of the probabilistic sensitivity analysis for the two interventions sNPWT and SC. Each point on the plot corresponds to one trial in the Monte-Carlo simulation (2000 simulations were conducted) comparing the incremental effectiveness and incremental costs of sNPWT compared to SC. Costs for sNPWT were consistently lower (read on the y-axis) and effectiveness highest (read on the x-axis) for the SSC prevention following CABG compared to SC. The figure therefore shows that sNPWT is cost-saving

We also performed a probabilistic sensitivity analysis, and presented the results as cost-effectiveness acceptability curves and the cost-effectiveness plane. The cost-effectiveness acceptability curves illustrate the probability that an intervention is cost-effective compared with the alternative, for a range of maximum monetary values that a decision-maker is willing to pay for a unit change in outcome in this case measured in QALYs. In our model, the cost-effectiveness acceptability curves demonstrates that sNPWT is 100% cost-effective for the willingness-to-pay threshold figure of €50,000 as shown in Fig. 2. The cost-effectiveness plane shows that both incremental cost and incremental QALY estimates are associated with little uncertainty as 99% of samples (red dots) are located in the South East (SE) quadrant where sNPWT is associated with less costs and better clinical outcomes as shown in Fig. 3.

### Subgroup analysis

A study by Olsen 2002 et al. [23] which considered the risk factors for deep and superficial SSI after CABG surgery indicates that the risk of deep chest SSI was associated with a combination of obesity and diabetes, whereas increased risk of superficial chest SSI was associated primarily with obesity. In our analysis we considered the sub-group of patients with obesity, diabetes and smoking. The risk of SSI was increased by more than threefold for patients with BMI > 30 while for diabetes and smoking it’s more than 2.5 fold. In these high risk patients, sNPWT was shown result in greater savings when compared to standard care in patients following CABG surgery. Table 5 shows the results of the sub-group analysis. Bigger savings are observed when patients with BMI ≥ 30 are prophylactically treated with sNPWT than with standard care dressings.

**Table 5:** Sub-group analysis results for sNPWT compared to standard care in patients following CABG surgery

Sub-group	Cost saving with sNPWT	Additional QALYs due to sNPWT	Reduction in complications due to sNPWT
BMI ≥ 30	€ 1586	0.1147	0.1507
Diabetes	€ 1370	0.096	0.1262
Smoking	€ 1298	0.0898	0.118

Abbreviations: *sNPWT* single use negative pressure wound therapy, *BMI* body mass index, *QALYs* quality adjusted life years

## Discussion

This study examined the cost-effectiveness analysis of sNPWT compared to standard of care dressings in preventing SSI for patients undergoing CABG. The results of the study suggest that treating patients undergoing CABG with sNPWT is cost-saving resulting better clinical outcomes (0.311 more QALYs) and cheaper overall, with cost savings of €586 per patient. The probability of sNPWT being cost-effective is 100%, indicating decision certainty and little chance of error in a decision based on this cost-effectiveness analysis.

A limited number of cost-effectiveness studies evaluating the use of sNPWT in closed incisions have been published [12, 13] and they conclude that negative pressure wound therapy is cost-effective. However, to our knowledge, there is no published data on cost-effectiveness analysis of sNPWT in preventing wound complications following CABG surgery. This may be explained by lack of clinical evidence to support sNPWT as we found out during the literature search. As noted earlier, we are aware of an ongoing clinical trial comparing sNPWT with standard care following CABG surgery (Dr Carlos Velasco; Hospital Juan Canalejo, Spain; personal communication).

The model adopted a number of conservative assumptions so the projected savings may actually under-estimate the true financial impact. For instance, we only captured SSI as an outcome and did not include other outcomes such as healing. Potentially infected wounds will take longer to heal and might develop into chronic wounds which are costly to treat [24]. Equally, dressing changes was not captured as an outcome, however we know based on previous studies that infected wounds would require more dressing changes making the model conservative favoring the strategy with more SSIs. For instance there were few dressing changes in the sNPWT group (*p* = 0.002) compared to standard care after hip and knee surgery, while there were more wounds with superficial infections in the standard care arm [11].

Given the diversity of health systems across the world, the results of this analysis should be interpreted with caution. We acknowledge that the reimbursement systems, relative prices, and treatment practices, are important issues that vary from country to country hence country-specific assumptions may be required. However, we note that our cost-effectiveness results were tested in sensitivity analysis and the RCT measured SSI in a standard way using the ECDC matrix. A study by Hansen 2012, found that there was a high degree of concordance between European and US case definitions of healthcare-associated infections [25]. Therefore, the measurement of SSI in a standard way suggests that the results of the RCT which drive the cost-effectiveness are likely to be replicated in setting with similar baseline risks. Furthermore, our model is based on clinical data that comes from a single center RCT, multi center trials should be preferred as they yield data which is easily generalisable. We therefore encourage other scholars to update the economic model once additional evidence becomes available.

## Conclusions

Our analysis found sNPWT to be less costly and more effective (dominant), resulting in an overall cost decrease of €586 per patient when used prophylactically in patients undergoing coronary artery by-pass surgery. These results remained stable in sensitivity analyses with bigger savings identified in sub-groups of patients with elevated risk of surgical site complications such as those patients with diabetes, obese and smokers. We however recommend that additional economic studies should be conducted as new evidence on the use of sNPWT in CABG patients becomes available to validate the results of this economic analysis.

## Abbreviations

BMI: Body mass index
CABG: Coronary artery bypass grafting
ECDC: European Centre for Disease Prevention and Control
QALY: Quality adjusted life years
RCT: Randomised controlled trial
sNPWT: Single use negative pressure wound therapy dressings
SSC: Surgical site complications
SSI: Surgical site infections

## References

[1] European Centre for Disease Prevention and Control (2013). Surveillance of surgical site infections in Europe 2010–2011.

[2] Gheorghe Adrian, Moran Grace, Duffy Helen, Roberts Tracy, Pinkney Thomas, Calvert Melanie (2015). Health Utility Values Associated with Surgical Site Infection: A Systematic Review. Value in Health.

[3] Reddy VS. Use of closed incision management with negative pressure therapy for complex cardiac patients. Cureus. 2016. 10.7759/cureus.506. 10.7759/cureus.506 PMC4807920 27026831

[4] Shepard John, Ward William, Milstone Aaron, Carlson Taylor, Frederick John, Hadhazy Eric, Perl Trish (2013). Financial Impact of Surgical Site Infections on Hospitals. JAMA Surgery.

[5] Dohmen PM, Gabbieri D, Weymann A, Linneweber J, Konertz W (2009). Reduction in surgical site infection in patients treated with microbial sealant prior to coronary artery bypass graft surgery: a case control study. J Hosp Infect.

[6] Graf Karolin, Ott Ella, Vonberg Ralf-Peter, Kuehn Christian, Schilling Tobias, Haverich Axel, Chaberny Iris Freya (2011). Surgical site infections—economic consequences for the health care system. Langenbeck's Archives of Surgery.

[7] Lee BY, Wiringa AE, Bailey RR, Goyal V, Lewis GJ, Tsui BYK (2010). Screening cardiac surgery patients for MRSA: an economic computer model. Am J Manag Care.

[8] Si D, Rajmokan M, Lakhan P, Marquess J, Coulter C, Paterson D. Surgical site infections following coronary artery bypass graft procedures: 10 years of surveillance data. BMC Infect Dis. 2014. 10.1186/1471-2334-14-318. 10.1186/1471-2334-14-318 PMC4061097 24916690

[9] Grauhan Onnen, Navasardyan Artashes, Hofmann Michael, Müller Peter, Stein Julia, Hetzer Roland (2013). Prevention of poststernotomy wound infections in obese patients by negative pressure wound therapy. The Journal of Thoracic and Cardiovascular Surgery.

[10] Jenks PJ, Laurent M, McQuarry S, Watkins R (2014). Clinical and economic burden of surgical site infection (SSI) and predicted financial consequences of elimination of SSI from an English hospital. J Hosp Infect.

[11] Karlakki S, Whittall C, Kuiper JH, Hamad AK (2016). Incisional negative pressure wound therapy dressings (iNPWTd) in routine primary hip and knee replacements – a randomised controlled trial. Bone Joint Res.

[12] Nherera Leo M., Trueman Paul, Karlakki Sudheer L. (2017). Cost-effectiveness analysis of single-use negative pressure wound therapy dressings (sNPWT) to reduce surgical site complications (SSC) in routine primary hip and knee replacements. Wound Repair and Regeneration.

[13] Tuffaha HW, Gillespie BM, Chaboyer W, Gordon LG, Scuffham PA (2015). Cost-utility analysis of negative pressure wound therapy in high-risk cesarean section wounds. J Surg Res.

[14] Strugala V, Martin R (2017). Meta-analysis of comparative trials evaluating a prophylactic single-use negative pressure wound therapy system for the prevention of surgical site complications. Surg Infect.

[15] Hyldig N., Birke-Sorensen H., Kruse M., Vinter C., Joergensen J. S., Sorensen J. A., Mogensen O., Lamont R. F., Bille C. (2016). Meta-analysis of negative-pressure wound therapy for closed surgical incisions. British Journal of Surgery.

[16] Cristofolini M., Worlitzsch D., Wienke A., Silber R.-E., Borneff-Lipp M. (2012). Surgical site infections after coronary artery bypass graft surgery: incidence, perioperative hospital stay, readmissions, and revision surgeries. Infection.

[17] Witt-Majchrzak A, Żelazny P, Snarska J. Preliminary outcome of treatment of postoperative primarily closed sternotomy wounds treated using negative pressure wound therapy. Pol Przegl Chir. 2015. 10.2478/pjs-2014-0082. 10.2478/pjs-2014-0082 25720104

[18] Germany Federal Statistical Office https://www.destatis.de/EN/FactsFigures/SocietyState/Population/Deaths/Deaths.html, (Accessed 15 Mar 2018).

[19] Wu Chuntao, Camacho Fabian T., Wechsler Andrew S., Lahey Stephen, Culliford Alfred T., Jordan Desmond, Gold Jeffrey P., Higgins Robert S.D., Smith Craig R., Hannan Edward L. (2012). Risk Score for Predicting Long-Term Mortality After Coronary Artery Bypass Graft Surgery. Circulation.

[20] The Aerzteblatt. https://www.aerzteblatt.de/archiv/43690/Krankenhaus-Management-Kompetenzzentren-sind-zukunftstraechtig, (Accessed 15 Mar 2018).

[21] Zeidler Jan, Mittendorf Thomas, Vahldiek Gunda, Graf von der Schulenburg J.-Matthias (2008). Kostenvergleichsanalyse der ambulanten und stationären kardiologischen Rehabilitation. Herz Kardiovaskuläre Erkrankungen.

[22] The Vdek association of health insurances. https://www.vdek.com/vertragspartner/heilmittel/rahmenvertrag/_jcr_content/par/download_19/file.res/Verg%C3%BCtungsvereinbarung_West_2016_UF_160321.pdf. (Accessed 15 Mar 2018).

[23] Olsen Margaret A., Lock-Buckley Patricia, Hopkins Diane, Polish Louis B., Sundt Thoralf M., Fraser Victoria J. (2002). The risk factors for deep and superficial chest surgical-site infections after coronary artery bypass graft surgery are different. The Journal of Thoracic and Cardiovascular Surgery.

[24] Posnett J, Franks PJ (2008). The burden of chronic wounds in the UK. Nurs Times.

[25] Hansen Sonja, Sohr Dorit, Geffers Christine, Astagneau Pascal, Blacky Alexander, Koller Walter, Morales Ingrid, Moro Maria, Palomar Mercedes, Szilagyi Emese, Suetens Carl, Gastmeier Petra (2012). Concordance between European and US case definitions of healthcare-associated infections. Antimicrobial Resistance and Infection Control.

